# Everyday Life of People Living with Dementia and its Impact on Their Lifestyle

**DOI:** 10.1002/alz.085831

**Published:** 2025-01-09

**Authors:** Istikhar Ali, Aparajit Ballav Dey

**Affiliations:** ^1^ Jawaharlal Nehru University, South Delhi, Delhi India; ^2^ Venu Geriatric Institute, South Delhi, Delhi India; ^3^ All India Institute of Medical Sciences, New delhi, India, New Delhi India

## Abstract

**Background:**

This ethnographic inquiry offers a nuanced exploration into the lives of individuals contending with dementia in India, utilizing data from the Harmonized Diagnostic Assessment of Dementia for the Longitudinal Aging Study in India (LASI‐DAD). Recognizing dementia as a complex global health challenge, we delve into its unique manifestations within India’s diverse regional and cultural landscape. While LASI‐DAD provides valuable quantitative insights into prevalence and risk factors, our research complements these statistics by providing an in‐depth understanding of the daily lives and lifestyles of those affected.

**Method:**

Employing purposive sampling, we meticulously captured diverse experiences across regions, languages, and socioeconomic backgrounds of individuals aged 60 and older. Through a blend of participant observation and in‐depth interviews, we systematically unraveled the multifaceted dimensions of living with dementia in India, exploring daily routines, coping strategies, and familial and societal dynamics.

**Result:**

Thematic analysis illuminated the profound realities encapsulated in the “Everyday Life of People Living with Dementia and Its Impact on Their Lives,” revealing two interconnected sub‐themes: 1. Emotional Struggles, where participants vividly described challenges adapting to contemporary settings and the emotional toll of living with dementia. 2. Evolving Social Dynamics and Behavioral Changes, wherein participants shared experiences of feeling marginalized in social settings amid the changes in their behavior and cognition.

This ethnographic inquiry extends beyond academic realms, carrying profound implications for individuals living with dementia and their families in India and globally. As the global dementia burden intensifies, our study contributes insights that foster more compassionate, culturally sensitive, and effective approaches to dementia care and management.

**Conclusion:**

This study serve as the foundation for a broad exploration of the multifaceted experiences of people living with dementia, these insights are crucial for developing empathetic and effective support systems. The integration of ethnographic methods with LASI‐DAD’s dataset in this study provides a regionally and culturally nuanced understanding of dementia in India. Our research also significantly informs local healthcare and support systems, enriching the global discourse on dementia and offering vital insights to enhance the quality of life for individuals living with dementia, their families, and caregivers, both in India and worldwide.

